# Activation of the hypoxia-inducible factor pathway by roxadustat improves glucose metabolism in human primary myotubes from men

**DOI:** 10.1007/s00125-024-06185-6

**Published:** 2024-05-30

**Authors:** Selina Mäkinen, Sreesha Sree, Tuulia Ala-Nisula, Henric Kultalahti, Peppi Koivunen, Heikki A. Koistinen

**Affiliations:** 1 Minerva Foundation Institute for Medical Research, Helsinki, Finland; 2 Department of Medicine, University of Helsinki and Helsinki University Hospital, Helsinki, Finland; 3 Biocenter Oulu, Faculty of Biochemistry and Molecular Medicine, Oulu Center for Cell-Matrix Research, University of Oulu, Oulu, Finland

**Keywords:** Glucose metabolism, Hypoxia-inducible factor, Insulin resistance, Insulin signalling, Primary human muscle cells, Roxadustat

## Abstract

**Aims/hypothesis:**

Hypoxia-inducible factor prolyl 4-hydroxylase (HIF-P4H) enzymes regulate adaptive cellular responses to low oxygen concentrations. Inhibition of HIF-P4Hs leads to stabilisation of hypoxia-inducible factors (HIFs) and activation of the HIF pathway affecting multiple biological processes to rescue cells from hypoxia. As evidence from animal models suggests that HIF-P4H inhibitors could be used to treat metabolic disorders associated with insulin resistance, we examined whether roxadustat, an HIF-P4H inhibitor approved for the treatment of renal anaemia, would have an effect on glucose metabolism in primary human myotubes.

**Methods:**

Primary skeletal muscle cell cultures, established from biopsies of *vastus lateralis* muscle from men with normal glucose tolerance (NGT) (*n*=5) or type 2 diabetes (*n*=8), were treated with roxadustat. Induction of HIF target gene expression was detected with quantitative real-time PCR. Glucose uptake and glycogen synthesis were investigated with radioactive tracers. Glycolysis and mitochondrial respiration rates were measured with a Seahorse analyser.

**Results:**

Exposure to roxadustat stabilised nuclear HIF1α protein expression in human myotubes. Treatment with roxadustat led to induction of HIF target gene mRNAs for *GLUT1* (also known as *SLC2A1*), *HK2*, *MCT4* (also known as *SLC16A4*) and *HIF-P4H-2* (also known as *PHD2* or *EGLN1*) in myotubes from donors with NGT, with a blunted response in myotubes from donors with type 2 diabetes. mRNAs for *LDHA*, *PDK1* and *GBE1* were induced to a similar degree in myotubes from donors with NGT or type 2 diabetes. Exposure of myotubes to roxadustat led to a 1.4-fold increase in glycolytic rate in myotubes from men with NGT (*p*=0.0370) and a 1.7-fold increase in myotubes from donors with type 2 diabetes (*p*=0.0044), with no difference between the groups (*p*=0.1391). Exposure to roxadustat led to a reduction in basal mitochondrial respiration in both groups (*p*<0.01). Basal glucose uptake rates were similar in myotubes from donors with NGT (20.2 ± 2.7 pmol mg^−1^ min^−1^) and type 2 diabetes (25.3 ± 4.4 pmol mg^−1^ min^−1^, *p*=0.4205). Treatment with roxadustat enhanced insulin-stimulated glucose uptake in myotubes from donors with NGT (1.4-fold vs insulin-only condition, *p*=0.0023). The basal rate of glucose incorporation into glycogen was lower in myotubes from donors with NGT (233 ± 12.4 nmol g^−1^ h^−1^) than in myotubes from donors with type 2 diabetes (360 ± 40.3 nmol g^−1^ h^−1^, *p*=0.0344). Insulin increased glycogen synthesis by 1.9-fold (*p*=0.0025) in myotubes from donors with NGT, whereas roxadustat did not affect their basal or insulin-stimulated glycogen synthesis. Insulin increased glycogen synthesis by 1.7-fold (*p*=0.0031) in myotubes from donors with type 2 diabetes. While basal glycogen synthesis was unaffected by roxadustat, pretreatment with roxadustat enhanced insulin-stimulated glycogen synthesis in myotubes from donors with type 2 diabetes (*p*=0.0345).

**Conclusions/interpretation:**

Roxadustat increases glycolysis and inhibits mitochondrial respiration in primary human myotubes regardless of diabetes status. Roxadustat may also improve insulin action on glycogen synthesis in myotubes from donors with type 2 diabetes.

**Graphical Abstract:**

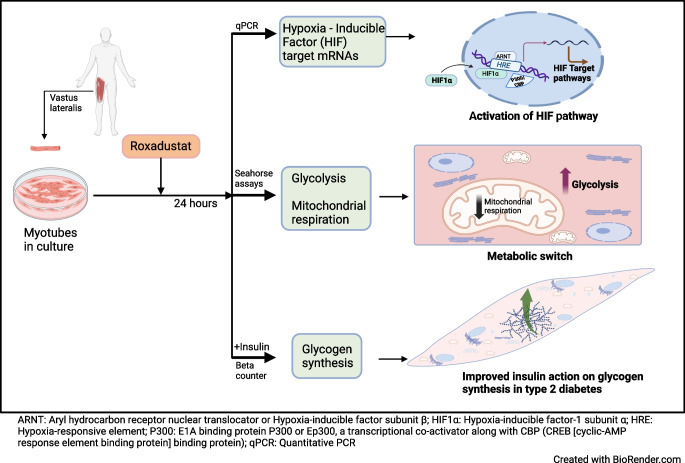

**Supplementary Information:**

The online version contains peer-reviewed but unedited supplementary material available at 10.1007/s00125-024-06185-6.


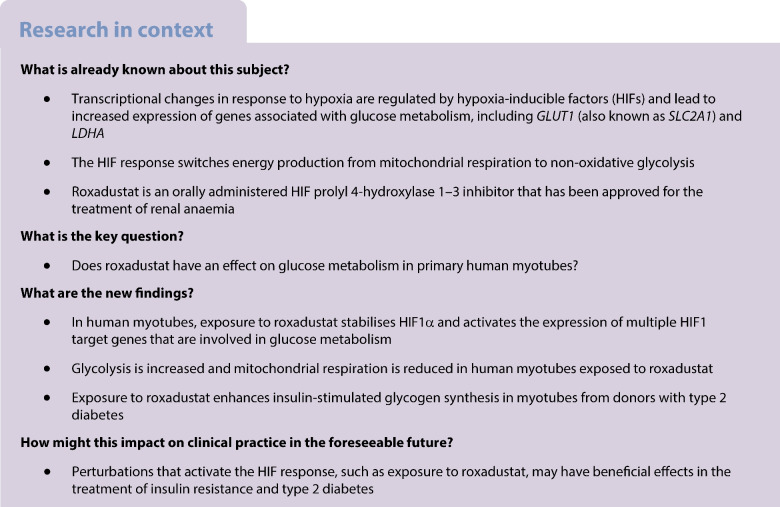


## Introduction

Activation of the hypoxia-inducible factor (HIF) pathway regulates cellular adaptation to hypoxia by affecting the expression of hundreds of target genes [1, 2]. This transcriptional response involves genes in various pathways such as those regulating erythropoiesis, angiogenesis, cell proliferation, apoptosis and glucose metabolism [3, 4]. Thus, proper functioning of the HIF pathway is essential for cell survival.

The transcription factor HIF1 is a heterodimer of two subunits: oxygen-regulated HIF1α; and constitutively expressed HIF1β/aryl hydrocarbon receptor nuclear translocator (ARNT) [4–6]. When oxygen levels are normal, HIF1α protein undergoes hydroxylation by HIF prolyl 4-hydroxylases (HIF-P4Hs, also known as prolyl hydroxylase domain proteins [PHDs] and egl-9 family hypoxia-inducible factors [EGLNs]) and is targeted to proteasomal degradation. Under hypoxia, HIF1α is stabilised, it translocates into the nucleus and dimerises with HIF1β/ARNT, initiating transcription of the responsive target genes [4–6]. For example, HIF1α is responsible for induction of glycolytic genes during hypoxia in order to support non-oxidative energy production [7].

Roxadustat (FG-4592) is an orally administered HIF-P4H 1–3 inhibitor that has been approved for the treatment of renal anaemia [8–10]. It reversibly inhibits three HIF-P4H isoenzymes, leading to the stabilisation of the HIFα subunits [11]. HIF binding to hypoxia-responsive elements (HREs) leads to increased expression of not only erythropoiesis-stimulating genes but also genes associated with glucose metabolism, including those encoding GLUT1 (*Glut1*, also known as *Slc2a1*) and lactate dehydrogenase A (*Ldha*) [12, 13]. Emerging preclinical and clinical data suggest that the activation of the hypoxia response through HIF-P4H inhibition may lead to improved metabolic health [10]. For example, in clinical trials of renal anaemia treatment, roxadustat decreased serum cholesterol and triglyceride concentrations and increased the HDL/LDL ratio [14]. HIF-P4H-2-deficient mice have improved glucose tolerance and insulin sensitivity, and oral administration of HIF-P4H inhibitor to wild-type mice improves insulin resistance and glucose intolerance during a high-fat diet [15].

Insulin resistance in skeletal muscle is a central and early defect in the pathogenesis of type 2 diabetes [16–18]. Therefore, interventions aiming at improving glucose metabolism in skeletal muscle are of interest in treating this metabolic disease. While preclinical data suggest improved insulin sensitivity in response to HIF-P4H inhibition [15], the effect of HIF pathway activation on glucose metabolism in human skeletal muscle is currently unknown. We are not aware of published studies with roxadustat that have assessed glucose metabolism in humans. Therefore, here we have used roxadustat to activate the HIF pathway and examined the effect of roxadustat on glucose metabolism in primary human myotubes from donors with normal glucose tolerance (NGT) or type 2 diabetes.

## Methods

### Reagents

Reagents are listed in the electronic supplementary material (ESM) Methods.

### Participants, muscle biopsies and isolation of primary muscle cells

We recruited men for muscle biopsies by advertisements (see Table 1 for the men’s characteristics). An OGTT with 75 g glucose was performed on a separate day to the muscle biopsy. Five men had NGT (WHO criteria) and were not using any regular medications. Five were newly diagnosed with type 2 diabetes and were not yet treated with glucose-lowering medications. Three men with a previous diagnosis of type 2 diabetes were recruited from outpatient clinics. They were treated with diet (*n*=1), with a combination of metformin and dipeptidyl peptidase-4 inhibitor (*n*=1), or with a combination of metformin, glucagon-like peptide-1 receptor agonist and insulin (*n*=1), and their duration of diabetes was 2, 9 and 15 years, respectively. Diabetes was classified as type 2 diabetes by excluding type 1 diabetes based on clinical history, and serum insulin and C-peptide concentrations (Table 1). Participants were representative of middle-aged Finnish men and self-identified as White. The studies were approved by The Ethical Committee of Department of Medicine, Helsinki University Hospital and were conducted according to the principles of the Declaration of Helsinki as revised in 2008. Participants gave written informed consent before participation.

**Table 1:** Clinical characteristics of participants

Characteristic	NGT	T2D	*p* value^a^
*N*	5	8	
Age, years	51.1 ± 4.9	58.2 ± 3.8	0.2751
Weight, kg	79.7 ± 4.5	99.9 ± 3.4	0.0039
Height, cm	180.5 ± 3.2	177.8 ± 2.3	0.5003
BMI, kg/m^2^	24.4 ± 0.9	31.8 ± 1.6	0.0057
Waist, cm	91 ± 2	112 ± 3	0.0007
Hip, cm	94 ± 2	105 ± 2	0.0053
WHR	0.97 ± 0.01	1.06 ± 0.02	0.0137
Fasting plasma glucose, mmol/l	5.5 ± 0.2	7.9 ± 0.7	0.0199
Fasting serum insulin, pmol/l^b^	38 ± 4	152 ± 27	0.0073
Fasting serum C-peptide, nmol/l	0.5 ± 0.04	1.3 ± 0.2	0.0085
HbA_1c_, mmol/mol	34.4 ± 0.8	46.7 ± 2.3	0.0019
HbA_1c_, %	5.3 ± 0.1	6.4 ± 0.2	0.0019
HOMA-IR	1.4 ± 0.2	7.7 ± 1.4	0.0058

Data are expressed as mean ± SEM

^a^Analysed by unpaired two-tailed Student’s *t* test

^b^Fasting serum insulin was collected in mU/l and converted to pmol/l using a conversion factor of 6.945, using the AMA Manual of Style 11th Edition: a guide for authors and editors SI conversion calculator (available from https://academic.oup.com/amamanualofstyle/si-conversion-calculator; accessed 13 March 2024)

T2D, type 2 diabetes

Primary muscle cell cultures were established as described previously [19]. Briefly, muscle biopsies were obtained, after participants had fasted overnight, under local anaesthesia (10 mg/ml lidocaine hydrochloride), from the *vastus lateralis* muscle. Myogenic satellite cells were isolated by trypsinisation and cultured in DMEM/F12 consisting of high-glucose (17.5 mmol/l) and high-serum (20% (vol./vol.) FBS) concentrations, supplemented with 1% (vol./vol.) penicillin-streptomycin and 1% (vol./vol.) amphotericin B. Primary myoblasts were purified from other cell types with CD56^+^-labelled magnetic beads. Myoblasts were differentiated into multinucleated myotubes in low-glucose (5.6 mmol/l) and low-serum (2% (vol./vol.) FBS) DMEM/F12 for 6 days.

### Pretreatment with roxadustat

Roxadustat (Cayman Chemicals, Ann Arbor, MI, USA) was dissolved in DMSO and used for myotubes treatment at a final concentration of 10 μmol/l (see ESM Methods). Preincubation with 0.1% (vol./vol.) DMSO acted as a control treatment. Pretreatments were conducted in serum-free (0% FBS) and low-glucose (5.6 mmol/l) DMEM/F12 supplemented with 0.5% (wt/vol.) fatty acid-free BSA. All incubations were performed at 37°C in a 5% CO_2_ incubator unless otherwise noted. The samples were not randomised.

### Western blot analyses

Expression of nuclear HIF1α protein, total protein expression and phosphorylation of insulin signalling targets Akt-Ser^473^, Akt substrate of 160 kDa (AS160)-Thr^642^ and glycogen synthase kinase 3β (GSK3β)-Ser^9^ were examined by western blotting (see ESM Methods). The samples included in the insulin signalling analyses were not masked.

### Quantitative real-time PCR analyses

For quantitative real-time PCR (qPCR) analyses, total RNA was isolated from pretreated myotubes with the E.Z.N.A. total RNA Kit I (Omega Bio-Tek, Norcross, GA, USA) and reverse transcribed with an iScript cDNA Synthesis Kit (Bio-Rad, Hercules, CA, USA). qPCR was then performed with iTaq SYBR Green Supermix with ROX (Bio-Rad) in a C1000 Touch Thermal Cycler and a CFX96 Touch Real-Time PCR Detection System (Bio-Rad). Primers are shown in ESM Table 1. The gene encoding TATA box binding protein (*TBP*) was used as a reference. The qPCR samples were masked.

### Metabolic assays

#### Glucose uptake

Glucose uptake was measured in triplicate by detecting the intracellular accumulation of 2-(1,2-[^3^H])deoxy-d-glucose (final sp. act. 3.7 GBq/mmol), as described [20] (see ESM Methods). Briefly, after pretreatment with 10 μmol/l roxadustat (or 0.1% DMSO as control) for 24 h, primary human myotubes were stimulated with or without 100 nmol/l insulin for 1 h, followed by the addition of radioactive glucose analogue for 20 min. Cytochalasin B (50 μmol/l) was used to subtract the non-specific glucose uptake [21]. Data (in pmol mg^−1^ min^−1^) were expressed as a fold over basal control sample of each participant. The samples included in the glucose uptake assays were not masked.

#### Glycogen synthesis

Glycogen synthesis was measured in triplicate by detecting d-[^14^C]glucose (final sp. act. 6.6 MBq/mmol) incorporation into glycogen [20] (see ESM Methods). Briefly, after pretreatment with 10 μmol/l roxadustat (or 0.1% DMSO as control) for 24 h, primary human myotubes were incubated with or without 100 nmol/l insulin together with radioactive glucose for 90 min. Data (in nmol g^−1^ h^−1^) were expressed as a fold over basal control sample of each participant. The samples included in the glycogen synthesis assays were not masked.

#### Glycolysis

Glycolysis was determined with Seahorse XFe96 analyser (Agilent Technologies, Santa Clara, CA, USA) in differentiated primary human myotubes under basal conditions using an XF Glycolytic Rate Assay kit (Agilent Technologies) (see ESM Methods). Glycolytic rate (proton efflux rate in pmol min^−1^ μg^−1^) was expressed as a fold over control sample of each participant. The samples included in the glycolysis assays were not masked.

### Mitochondrial respiration

Mitochondrial oxygen consumption rate (OCR) was measured with a Seahorse XFe96 analyser (Agilent Technologies) in differentiated primary human myotubes under basal conditions using XF Mito Stress Test Assay kit (Agilent Technologies) (see ESM Methods). Data (in pmol min^−1^ μg^−1^) were expressed as a fold over control sample of each participant. The samples included in the mitochondrial OCR assays were not masked.

### Other determinations

The circumference of the waist was measured to the nearest 0.5 cm midway between the lower rib margin and the iliac crest and the circumference of the hip was measured at the level of the trochanters with a soft measuring tape [22, 23]. Plasma glucose concentration was analysed by the hexokinase method, HbA_1c_ was determined by an immunological method, and fasting serum insulin and C-peptide concentrations were determined by chemiluminescence immunoassays at HUSLAB (Central laboratory of the Helsinki University Hospital, Hospital District of Helsinki and Uusimaa). Insulin resistance was estimated by HOMA-IR [24, 25].

### Statistical analyses

Data are presented as mean ± SEM. Statistical analyses were performed using GraphPad Prism (version 10.1.0 for Mac OS, www.graphpad.com, GraphPad Software, Boston, MA, USA). The normality of the data distribution was tested with the Shapiro–Wilk test. Two-way ANOVA with repeated measurements, followed by Holm–Šídák’s post hoc test for multiple comparisons, was used to analyse data unless otherwise noted. *p*<0.05 was considered statistically significant.

## Results

### Clinical characteristics of the participants

Primary muscle cell cultures were established from five men with NGT and eight men with type 2 diabetes (Table 1). Men with type 2 diabetes had higher BMI, fasting plasma glucose, serum insulin and C-peptide concentrations, HbA_1c_ and HOMA-IR than men with NGT. Their overall glycaemic control was good (Table 1). We first set out to study metabolic responses (glucose uptake, glycogen synthesis, glycolytic rate, mitochondrial respiration) in myotubes from five donors with NGT and five donors with type 2 diabetes. When analysing the glycogen synthesis results, myotubes from two donors with type 2 diabetes showed a robust response (2.0- and 2.5-fold) to insulin stimulation (see Glucose metabolism, below). Assays of glucose uptake and glycogen synthesis were performed in additional myotubes from three donors with type 2 diabetes, where the glycogen synthesis response to insulin was robust (2.4-fold) in myotubes from one donor and blunted in two donors with type 2 diabetes. Hence, the total number of myotubes from donors with type 2 diabetes was eight in assays of glucose uptake and glycogen synthesis. In other assays (qPCR, glycolytic rate, mitochondrial respiration), the five initial myotubes from donors with type 2 diabetes (three with a low insulin response and two with a robust insulin response in glycogen synthesis assays) were used.

### Roxadustat activates the HIF pathway and induces target gene expression in human myotubes

In initial time course studies, human myotubes were treated with 10 μmol/l roxadustat for 6 h, 24 h and 48 h (ESM Figs 1, 2). Incubation with 0.1% DMSO acted as a control treatment. Stabilisation of nuclear HIF1α protein was detected already at 6 h and was maintained up to 48 h (ESM Fig. 1). qPCR assays revealed the most prominent induction of HIF target gene mRNAs at 24 h (ESM Fig. 2). Based on these data, pretreatment for 24 h was chosen for further experiments.

**Fig. 1:**
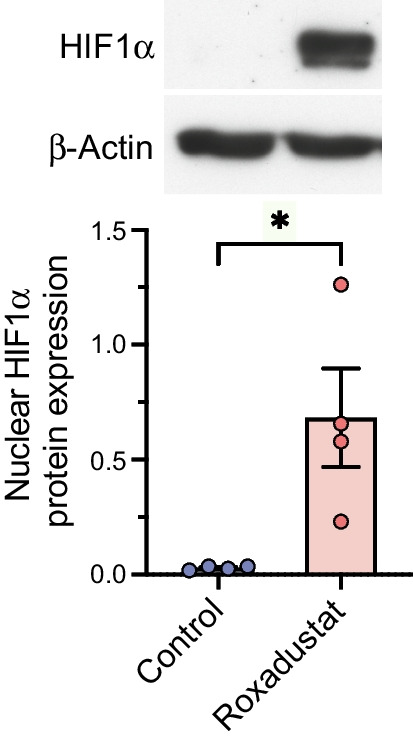
Nuclear HIF1α protein stabilisation by roxadustat in human myotubes. Primary human myotubes from men with NGT (*n*=4) were exposed to 10 μmol/l roxadustat or 0.1% DMSO as control for 24 h. Protein expression was analysed by western blotting. The quantified intensity of nuclear HIF1α protein was normalised to β-actin. Data are expressed as mean ± SEM. *p<0.05, analysed by paired one-tailed Student’s *t* test

**Fig. 2:**
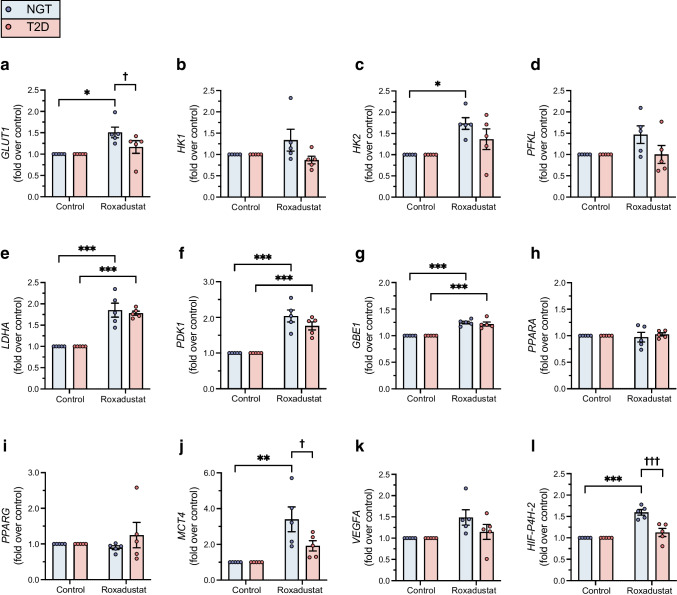
HIF target gene mRNA expression in roxadustat-treated myotubes. Primary human myotubes from men with NGT (*n*=5) or type 2 diabetes (*n*=5, consisting of three men with a low insulin response and two men with a robust insulin response in glycogen synthesis assays) were exposed to 10 μmol/l roxadustat or 0.1% DMSO as control for 24 h. qPCR was used to detect the induction of HIF-responsive genes. Values were normalised to the control sample for each individual. T2D, type 2 diabetes. Data are expressed as mean ± SEM. *p<0.05, **p<0.01, ***p<0.001 for roxadustat vs control; ^†^*p*<0.05, ^†††^*p*<0.001 for type 2 diabetes vs NGT; analysed by two-way ANOVA with repeated measurements, with Holm–Šídák’s post hoc test

Treatment of myotubes from four men with NGT with roxadustat for 24 h led to stabilisation of nuclear HIF1α protein (Fig. 1). Next, we examined the effect of 24 h roxadustat pretreatment on gene expression. There were no significant differences in the basal expression of target gene mRNAs in myotubes from donors with NGT and type 2 diabetes (ESM Fig. 3). To detect potential differences in response to roxadustat between the study groups, the expression of target gene mRNAs relative to the control sample for each participant was analysed (Fig. 2). Roxadustat exposure led to induction of HIF target gene mRNAs for *GLUT1* (Fig. 2a), *HK2* (encoding hexokinase 2) (Fig. 2c), *MCT4* (encoding monocarboxylate transporter 4) (Fig. 2j) and *HIF-P4H-2* (also known as *PHD2 or EGLN1*) (Fig. 2l) in myotubes from donors with NGT, with a blunted response in myotubes from donors with type 2 diabetes (Fig. 2a, j, l). HIF target gene mRNAs for *LDHA* (encoding lactate dehydrogenase A) (Fig. 2e)*, PDK1* (encoding pyruvate dehydrogenase kinase isoform 1) (Fig. 2f) and *GBE1* (encoding glycogen-branching enzyme 1) (Fig. 2g) were induced similarly in response to roxadustat in myotubes when comparing the NGT and type 2 diabetes groups. Other examined HIF target gene mRNAs, such as *HK1* (encoding hexokinase 1) (Fig. 2b), *PFKL* (encoding phosphofructokinase L) (Fig. 2d), *PPARA* (encoding peroxisome proliferator-activated receptor α) (Fig. 2h) and *PPARG* (encoding peroxisome proliferator-activated receptor γ) (Fig. 2i), or *VEGFA* (encoding vascular endothelial growth factor A) (Fig. 2k), were not significantly induced by roxadustat in either group.

### Glucose metabolism

#### Roxadustat enhances insulin action on glucose uptake into myotubes

**Fig. 3:**
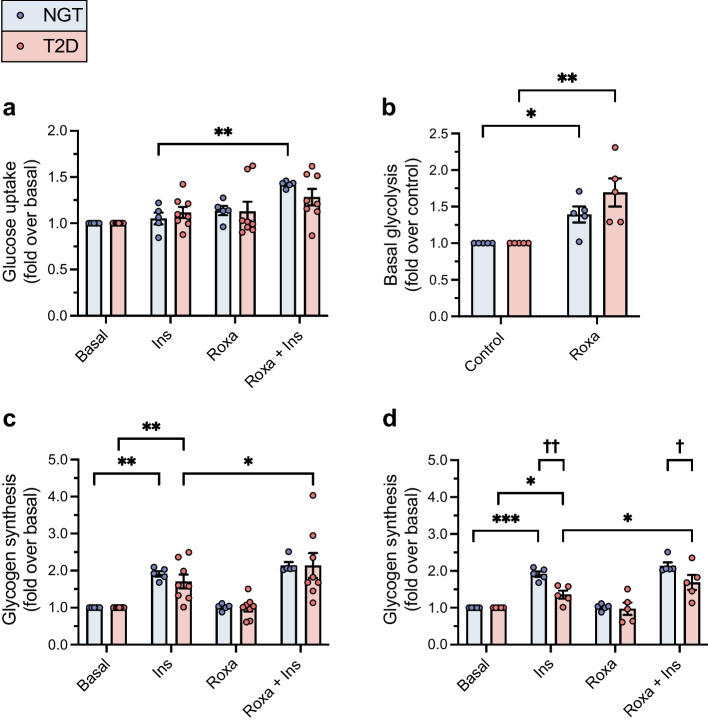
The effect of roxadustat on glucose metabolism in human myotubes. Primary human myotubes were exposed to 10 μmol/l roxadustat or 0.1% DMSO as control for 24 h, followed by metabolic tests with or without stimulation with 100 nmol/l insulin. (**a**) Basal and insulin-stimulated glucose uptake rate (in pmol mg^−1^ min^−1^) was determined in myotubes from men with NGT (*n*=5) or type 2 diabetes (*n*=8). (**b**) Basal glycolytic rate (in pmol min^−1^ μg^−1^) was determined in myotubes from men with NGT (*n*=5) or type 2 diabetes (*n*=5, consisting of three men with a low insulin response and two men with a robust insulin response in glycogen synthesis assays). (**c**) Basal and insulin-stimulated rates of glucose incorporation into glycogen (in nmol g^−1^ h^−1^) were determined in myotubes from men with NGT (*n*=5) or type 2 diabetes (*n*=8). (**d**) Basal and insulin-stimulated rates of glucose incorporation into glycogen (in nmol g^−1^ h^−1^) were determined in myotubes from men with NGT (*n*=5) and in a subgroup of myotubes that were more insulin-resistant in vitro, obtained from men with type 2 diabetes (*n*=5). Values were normalised to the basal (**a**, **c**, **d**) or control (**b**) sample of each participant. Ins, insulin; Roxa, roxadustat; T2D, type 2 diabetes. Data are expressed as mean ± SEM. *p<0.05, **p<0.01, ***p<0.001 for roxadustat, a combination of roxadustat and insulin or insulin only vs respective basal, control or insulin only; ^†^*p*<0.05, ^††^*p*<0.01 for type 2 diabetes vs NGT; analysed by two-way ANOVA with repeated measurements, with Holm–Šídák’s post hoc test

Basal glucose uptake rate was similar in myotubes from donors with NGT (20.2 ± 2.7 pmol mg^−1^ min^−1^) and type 2 diabetes (25.3 ± 4.4 pmol mg^−1^ min^−1^; *p*=0.4205, Student’s unpaired *t* test). Exposure to roxadustat did not modify basal glucose uptake in myotubes from either group. Pretreatment with roxadustat significantly enhanced insulin-stimulated glucose uptake into myotubes from donors with NGT (1.4-fold vs insulin-only condition; *p*=0.0023). This induction was not statistically significant in myotubes from donors with type 2 diabetes (1.2-fold; *p*=0.1230) (Fig. 3a).

#### Roxadustat increases glycolysis

The glycolytic rate was not significantly different in myotubes from donors with NGT (9.2 ± 1.6 pmol min^−1^ μg^−1^) vs type 2 diabetes (5.2 ± 1.1 pmol min^−1^ μg^−1^; *p*=0.0696, Student’s unpaired *t* test). Pre-exposure to roxadustat led to a significant increase in glycolytic rate in myotubes from donors with NGT (1.4-fold; *p*=0.0370) and myotubes from donors with type 2 diabetes (1.7-fold; *p*=0.0044). The increase in glycolysis in response to roxadustat was similar in myotubes from both groups (*p*=0.1391) (Fig. 3b).

#### Roxadustat enhances insulin action on glycogen synthesis in myotubes from donors with type 2 diabetes

The basal rate of glucose incorporation into glycogen was lower in myotubes from donors with NGT (233 ± 12.4 nmol g^−1^ h^−1^) than in myotubes from donors with type 2 diabetes (360 ± 40.3 nmol g^−1^ h^−1^; *p*=0.0344, Student’s unpaired *t* test). Insulin increased glycogen synthesis 1.9-fold, from 1.7 to 2.1-fold (*p*=0.0025), in myotubes from donors with NGT, whereas roxadustat did not affect basal or insulin-stimulated glycogen synthesis (Fig. 3c, d). In myotubes from donors with type 2 diabetes, insulin increased glycogen synthesis by 1.7-fold, from 1.02- to 2.5-fold (*p*=0.0031) (Fig. 3c). While basal glycogen synthesis was unaffected by roxadustat, insulin-stimulated glycogen synthesis was enhanced by roxadustat pretreatment in myotubes from donors with type 2 diabetes (*p*=0.0345), to levels observed in myotubes from donors with NGT (Fig. 3c).

Although all eight men with type 2 diabetes displayed a significantly higher HOMA-IR than men with NGT, suggesting the presence of insulin resistance (Table 1), myotubes from three men with type 2 diabetes responded surprisingly well to insulin, as their glycogen synthesis was increased by 2.0- to 2.5-fold. Therefore, we also performed a statistical analysis of glycogen synthesis assay data in the subgroup of five men with type 2 diabetes with more in vitro insulin-resistant myotubes. Here, insulin increased glycogen synthesis by 1.4-fold (*p*=0.0296) in myotubes from donors with type 2 diabetes, a response significantly reduced compared with that in myotubes from donors with NGT (*p*=0.0044). Roxadustat also improved the insulin-stimulated glycogen synthesis in this subgroup of myotubes from donors with type 2 diabetes (1.3-fold increase vs insulin-only condition) (*p*=0.0296) (Fig. 3d).

### Roxadustat decreases mitochondrial respiration

**Fig. 4:**
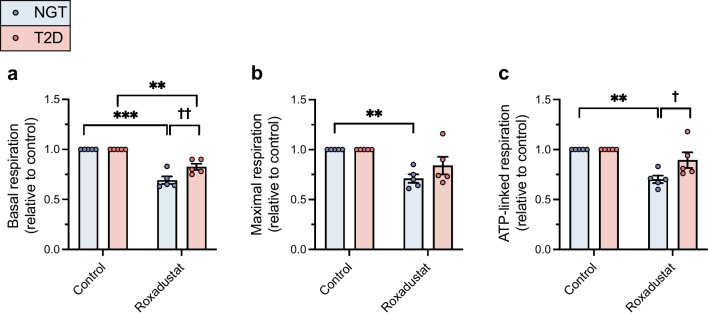
Mitochondrial respiration in roxadustat-treated myotubes. Primary human myotubes from men with NGT (*n*=5) or type 2 diabetes (*n*=5, consisting of three men with a low insulin response and two with a robust insulin response in glycogen synthesis assays) were exposed to 10 μmol/l roxadustat or 0.1% DMSO as control for 24 h. (**a**–**c**) Basal respiration (**a**), maximal respiration (**b**) and ATP-linked respiration (**c**) (in pmol min^−1^ μg^−1^) were analysed. Values were normalised to the control sample for each participant. T2D, type 2 diabetes. Data are expressed as mean ± SEM. **p<0.01, ***p<0.001 for roxadustat vs control; ^†^*p*<0.05, ^††^*p*<0.01 for type 2 diabetes vs NGT; analysed by two-way ANOVA with repeated measurements, with Holm–Šídák’s post hoc test

Basal, maximal and ATP-linked respiration were similar in myotubes from donors with NGT (in pmol min^−1^ μg^−1^: basal 4.0 ± 0.4; maximal 7.0 ± 0.5; and ATP-linked 3.8 ± 0.4) and type 2 diabetes (in pmol min^−1^ μg^−1^: basal 3.6 ± 0.6 [*p*=0.5431, unpaired Student’s *t* test]; maximal 5.7 ± 0.7 [*p*=0.1604, unpaired Student’s *t* test]; and ATP-linked respiration 3.4 ± 0.4 [*p*=0.5282, unpaired Student’s *t* test]). Exposure to roxadustat led to a significant decrease in basal mitochondrial respiration in both myotubes from donors with NGT (*p*<0.001**)** and myotubes from donors with type 2 diabetes (*p*<0.01) (Fig. 4a). Roxadustat-induced reduction in basal respiration was slightly blunted in myotubes from donors with type 2 diabetes as compared with myotubes from donors with NGT (*p*=0.0025) (Fig. 4a). Exposure to roxadustat led also to a significant decrease in maximal respiration in myotubes from donors with NGT (*p*=0.0062). The decrease in maximal respiration in response to roxadustat was not statistically significant in myotubes from donors with type 2 diabetes (*p*=0.0503) (Fig. 4b), and the difference in the reduction in maximal respiration did not differ significantly between the phenotypes (*p*=0.1533) (Fig. 4b). Exposure to roxadustat led to a significant decrease in ATP-linked respiration in myotubes from donors with NGT (*p*=0.0025) (Fig. 4c). In myotubes from donors with type 2 diabetes, the decrease in ATP-linked respiration was not statistically significant (*p*=0.1215) and the difference in reduction of ATP-linked respiration in response to roxadustat was statistically significant between the study groups (*p*=0.0127) (Fig. 4c).

### Roxadustat and the insulin signalling pathway

**Fig. 5:**
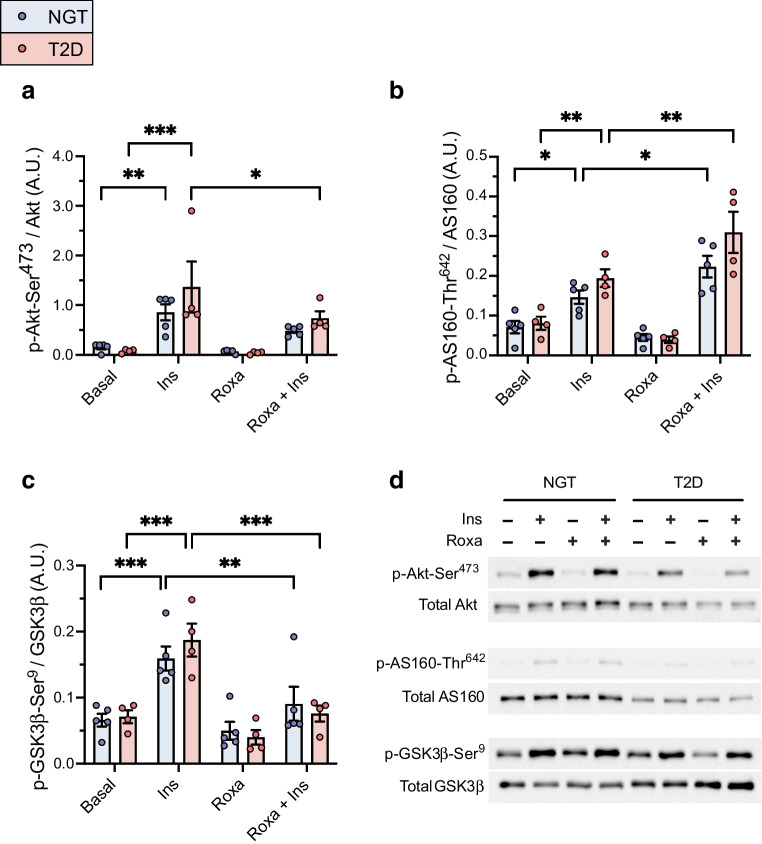
Analysis of activation of the insulin signalling pathway in roxadustat-treated myotubes. Primary human myotubes from men with NGT (*n*=5) or with type 2 diabetes (*n*=4, consisting of two men with a low insulin response and two men with a robust insulin response in glycogen synthesis assays) were pretreated with 10 μmol/l roxadustat or 0.1% DMSO as control for 24 h, followed by 10 min stimulation with 100 nmol/l insulin. Insulin signalling was analysed by western blotting. (**a**–**c**) Quantification of p-Akt-Ser^473^ (**a**), p-AS160-Thr^642^ (**b**) and p-GSK3β-Ser^9^ (**c**) was normalised to the intensity of their corresponding total proteins. (**d**) Representative images of the western blots. A.U., arbitrary units; Ins, insulin; Roxa, roxadustat; T2D, type 2 diabetes. Data are expressed as mean ± SEM. *p<0.05, **p<0.01, ***p<0.001 vs stimulation with insulin only, analysed by two-way ANOVA with repeated measurements, with Holm–Šídák’s post hoc test

Insulin stimulation led to increased phosphorylation of Akt-Ser^473^, AS160-Thr^642^ and GSK3β-Ser^9^ in myotubes from both donors with NGT and type 2 diabetes (Fig. 5a–d).

In myotubes from donors with NGT, exposure to roxadustat led to an increase in insulin-stimulated phosphorylation of AS160-Thr^642^, whereas insulin-stimulated phosphorylation of GSK3β-Ser^9^ was reduced. Exposure to roxadustat led to a reduction in insulin-stimulated phosphorylation of Akt-Ser^473^ and GSK3β-Ser^9^, and an increase in insulin-stimulated phosphorylation of AS160-Thr^642^ in myotubes from donors with type 2 diabetes (Fig. 5a–d).

## Discussion

Here, we tested whether pharmacological activation of the HIF pathway by roxadustat had an effect on glucose metabolism in human myotubes. Muscle cell cultures were established from healthy men as well as men with type 2 diabetes. Pretreatment with roxadustat led to an increase in glycolytic rate and a reduction in mitochondrial respiration in myotubes from both donors with NGT and donors with type 2 diabetes. While the effect of roxadustat was mainly neutral with respect to basal glucose uptake or glycogen synthesis, co-incubation with roxadustat enhanced insulin-stimulated glucose uptake into myotubes from donors with NGT and enhanced insulin-stimulated glycogen synthesis in myotubes from donors with type 2 diabetes.

The small molecule pan-HIF-P4H inhibitor roxadustat has been developed and recently accepted for treatment of renal anaemia [8–10]. However, roxadustat also demonstrates important effects on metabolism. Clinical trials in individuals with renal anaemia treated with roxadustat have reported improvements in lipid metabolism such as a decrease in serum cholesterol and triglyceride concentrations [14, 26]. Moreover, preclinical studies have revealed that genetic or pharmacological inhibition of HIF-P4H improves glucose tolerance and insulin sensitivity [15]. Thus, activation of the hypoxia response pathway by inhibiting HIF-P4Hs may be a promising novel way to treat metabolic diseases such as insulin resistance and type 2 diabetes.

HIF1 regulates the expression of genes involved in the cellular response to hypoxia [1, 2], such as those encoding angiogenic growth factors and glycolytic enzymes [10]. Exposure to roxadustat led to an increased mRNA expression of HIF target genes *GLUT1*, *HK2*, *MCT4* and *HIF-P4H-2* in myotubes from donors with NGT whereas this response was blunted in myotubes from donors with type 2 diabetes. The expression of mRNAs for *LDHA*, *PDK1* and *GBE1* were induced similarly in response to roxadustat in myotubes from both groups. Thus, roxadustat induced the expression of HIF1 target genes in human myotubes, with some differences noted depending on glucose tolerance status.

As skeletal muscle insulin resistance is a central and early defect in the pathogenesis of type 2 diabetes [16–18], detailed knowledge of the factors regulating insulin action in muscle is important and may provide novel insight into improving treatment of people with insulin resistance. Therefore, we tested whether roxadustat affects glucose metabolism in human skeletal muscle cells. Basal glucose uptake and basal glycogen synthesis were not affected by roxadustat in myotubes from individuals with either NGT or type 2 diabetes. In contrast, pretreatment with roxadustat led to increased insulin-stimulated glucose uptake into myotubes from donors with NGT. This difference was not statistically significant in myotubes from donors with type 2 diabetes due to variable responses. Neither basal nor insulin-stimulated glycogen synthesis were affected by roxadustat in myotubes from donors with NGT. However, insulin-stimulated glycogen synthesis was enhanced by roxadustat in myotubes from donors with type 2 diabetes. Interestingly, insulin’s action on glycogen synthesis was robust in three men with type 2 diabetes. Therefore, we analysed the glycogen synthesis data using only the more insulin-resistant myotubes, from five donors with type 2 diabetes. This yielded similar results and showed that roxadustat improved insulin-stimulated glycogen synthesis in this subgroup. Exposure to roxadustat reduced insulin-stimulated phosphorylation of Akt-Ser^473^ in myotubes from donors with type 2 diabetes and insulin-stimulated phosphorylation of GSK3β-Ser^9^ in myotubes from both groups. In contrast, insulin-stimulated phosphorylation of AS160-Thr^642^ was improved in response to roxadustat in both study groups, and this may have contributed to the observed effects in glucose metabolism. These data suggest that roxadustat enhances AS160 signalling and glucose metabolism in an Akt- and GSK3β-independent fashion. These mechanisms require further investigation.

During hypoxaemia, reprogramming of glucose metabolism takes place by HIF1-dependent induction of glycolytic genes, as well as HIF1-mediated suppression of mitochondrial oxidative phosphorylation, to promote cell survival by directing glucose metabolites from the mitochondria to glycolysis; this maintains ATP production during oxygen deprivation and prevents generation of harmful reactive oxygen species [4, 27–29]. Inhibition of mitochondrial OCR occurs via increased PDK1, which inhibits pyruvate dehydrogenase and prevents use of pyruvate in the mitochondrial citric acid cycle [29]. Our data agree with these observations. Exposure to roxadustat led to a significant increase in *PDK1* mRNA expression, activation of glycolysis and a reduction in mitochondrial respiration in myotubes from both study groups. Interestingly, the reduction of basal mitochondrial respiration in response to roxadustat was significantly greater in myotubes from donors with NGT than in those from donors with type 2 diabetes, suggesting differential regulation of the metabolic switch in myotubes from donors with type 2 diabetes. Consistent with this, exposure to roxadustat led to a reduction of mitochondrial ATP-linked respiration in myotubes from donors with NGT, whereas myotubes from donors with type 2 diabetes were not affected similarly. Taken together, these data show that HIF pathway activation by roxadustat leads to reprogramming of glucose metabolism in human myotubes. While overall the effects were similar in myotubes from men with NGT or type 2 diabetes, there were subtle differences in metabolic responses to roxadustat in skeletal muscle between the study groups, meriting future studies to analyse factors contributing to these differences.

While the debate as to whether the decrease in mitochondrial oxidative metabolism in skeletal muscle is the root cause of, or rather consequent to, insulin resistance and altered/dysregulated metabolism in diabetes is ongoing [30–33], the presence of skeletal muscle mitochondrial dysfunction in insulin resistance and type 2 diabetes is well established [34]. Therefore, it seems counterintuitive that perturbations that lead to a switch from oxidative to glycolytic metabolism would be beneficial in treating insulin resistance. As one of the hallmarks of insulin resistance is impaired glycogen synthesis in skeletal muscle during in vivo insulin stimulation [17, 18, 35], our observation that roxadustat treatment enhanced insulin action on glycogen synthesis in myotubes from donors with type 2 diabetes is exciting. It would be interesting to study the effects of roxadustat on glucose metabolism in vivo in people with insulin resistance. No serious adverse events have been reported for roxadustat in the treatment of anaemia of chronic kidney disease [9, 14]. However, pleotropic effects of the activation of the HIF pathway via HIF-P4H inhibition may also lead to undesired phenomena such as fibrosis, inflammation and tumour growth in some situations [36, 37]. More research on the molecular mechanisms and long-term clinical trials in indications beyond anaemia are needed for the confirmation of safety and efficacy of HIF-P4H inhibitors.

The main advantage of the current study is the use of human primary muscle cells derived from carefully characterised men with NGT or type 2 diabetes, thus providing a more accurate representation of the metabolic effects of the HIF pathway activation in human muscle cells compared with immortalised cell lines. A weakness of the study is that we only have primary muscle cell cultures established from male donors, excluding the possibility of analysing the effect of sex on the responses. Moreover, the sample size per group was limited, thus increasing the potential donor-to-donor variability and sensitivity to outliers. To overcome issues with interindividual variation, each participant acted as his own control, thus enabling the detection of potential differences in individual relative responses. The insulin response in the glycogen synthesis assay was robust in three donors with type 2 diabetes, despite their increased HOMA-IR. This suggests heterogeneity of insulin responses in different target tissues among people with type 2 diabetes and is in line with the increasing evidence of heterogeneity of type 2 diabetes [38, 39]. As HOMA-IR is reflective of hepatic insulin resistance [24, 25], our data suggest the presence of liver insulin resistance and normal insulin action in myotubes in these three men with type 2 diabetes.

In conclusion, exposure of human myotubes to roxadustat activates the HIF pathway and leads to reprogramming of glucose metabolism. Glycolysis is increased and mitochondrial oxygen consumption decreased in myotubes treated with roxadustat. Roxadustat treatment improved insulin action on glycogen synthesis in myotubes from donors with type 2 diabetes, suggesting that perturbations affecting the HIF pathway may be a novel way to improve metabolism in insulin-resistant individuals.

## Supplementary Information

Below is the link to the electronic supplementary material.

ESM 1 (PDF 1649 KB)

## Data Availability

The data that support the findings of this study are not openly available due to reasons of sensitivity.

## Abbreviations

ARNT: Aryl hydrocarbon receptor nuclear translocator
AS160: Akt substrate of 160 kDa
GSK3β: Glycogen synthase kinase 3β
HIF: Hypoxia-inducible factor
HIF-P4H: HIF prolyl 4-hydroxylase
NGT: Normal glucose tolerance
OCR: Oxygen consumption rate
PDK1: Pyruvate dehydrogenase kinase isoform 1
qPCR: Quantitative real-time PCR
