# Supplementary material for: Activation of the hypoxia-inducible factor pathway by roxadustat improves glucose metabolism in human primary myotubes from men
Source: Diabetologia. 2024 May 30;67(9):1943–54. doi: 10.1007/s00125-024-06185-6 (PMC11410918; doi:10.1007/s00125-024-06185-6)
Supplement: Supplementary file 1 — ESM 1 (PDF 1649 KB) [file 125_2024_6185_MOESM1_ESM.pdf]

ELECTRONIC SUPPLEMENTARY MATERIAL

Activation of the hypoxia-inducible factor pathway by roxadustat improves glucose metabolism in human primary myotubes from men

Selina Mäkinen[1,2]*, Sreesha Sree[1,2]*, Tuulia Ala-Nisula[3], Henric Kultalahti[1,2], Peppi Koivunen[3], Heikki A. Koistinen[1,2†]

*Selina Mäkinen and Sreesha Sree contributed equally to this study.

[1]Minerva Foundation Institute for Medical Research, Helsinki, Finland;

[2]Department of Medicine, University of Helsinki and Helsinki University Hospital, Helsinki, Finland

[3]Biocenter Oulu, Faculty of Biochemistry and Molecular Medicine, Oulu Center for Cell-Matrix Research, University of Oulu, Oulu, Finland

ESM Methods, pages 2-5

ESM Table 1, page 6

ESM Figures, pages 7-9

References, page 10

ESM Methods

List of reagents

Cell culture medias were from Gibco (Grand Island, NY, USA): high-glucose DMEM/F12 (3150 mg/l, 17.5 mmol/l, cat. 31331), low-glucose DMEM/F12 (1000 mg/l, 5.6 mmol/l, cat. 21885), glucose-free DMEM (cat. 11966).

Seahorse reagents and consumables were from Agilent Technologies Inc (Santa Clara, CA, USA): XF Mito Stress Test Kit, XF Glycolytic Rate Assay Kit, XF DMEM medium pH 7.4, XF 1.0 mol/l Glucose Solution, XF 100 mmol/l Pyruvate Solution, XF 200 mmol/l Glutamine Solution, Seahorse XFe96 plates and sensor cartridges.

Other reagents: MACS $CD56^{+}$ human MicroBeads were from Miltenyi Biotec (Auburn, CA, USA) Amphotericin B (Fungizone) was from Gibco (Grand Island, NY, USA). Penicillin-streptomycin solution, FBS, 2-deoxy-D-glucose, rat liver glycogen carrier, and cytochalasin B, were from Sigma (St Louis, MO, USA). Fatty acid-free BSA was from Biowest (Nuaillé, FR). Roxadustat was from Cayman Chemicals (Ann Arbor, MI, USA). Actrapid Insulin was from Novo Nordisk (Bagsværd, DK). Radioactive 2-(1,2-[^{3}H])deoxy-D-glucose and D-[^{14}C]glucose were from Perkin Elmer (Boston, MA, USA). Pierce BCA Protein Assay Kit, NE-PER Nuclear Extraction kit (cat. 78835) and ECL2 Western Blotting Substrate Kit were from ThermoFisher Scientific (Waltham, MA, USA). Complete Mini EDTA-free protease inhibitor cocktail tablets and PhosSTOP phosphatase inhibitor tablets were from Roche/Merck (Darmstadt, DE). Bradford assay, and Clarity Max Western ECL substrate were from Bio-Rad (Hercules, CA, USA).

Pretreatment with roxadustat

Roxadustat (Cayman Chemicals, Ann Arbor, MI, USA) was dissolved in DMSO and used on myotubes in a final concentration of 10 μmol/l. Preincubation with 0.1% (vol/vol) DMSO acted as a control treatment. Based on published literature [1, 2], and our own unpublished experience, roxadustat stabilizes HIF1α in concentrations between 5 to 50 μM. Concentrations above 20 μM are sometimes associated with increased cell death. Therefore, we decided to use 10 μM roxadustat, which stabilized HIF1α while no cell death was observed. Pretreatments were conducted in serum-free (0% FBS) and low-glucose (5.6 mmol/l) DMEM/F12 supplemented with 0.5% (wt/vol) fatty acid-free BSA. All incubations were performed at 37°C in a 5% CO_2 incubator unless otherwise noted.

Western blot analysis

Western blot analysis of the nuclear HIF1α stabilization was performed. Myotubes were lysed and nuclear fractions were extracted with a NE-PER Nuclear Extraction kit. Protein content was measured with the Bradford assay. Nuclear fractions were separated by SDS-PAGE, blotted onto PVDF membrane (cat. IPVH00010, Merck Millipore, Darmstadt, DE), and probed with primary antibodies against HIF1α (cat. 610959, 1:1000, BD Transduction Laboratories, Franklin Lakes, NJ, USA), followed by HRP-conjugated secondary antibody Polyclonal Goat Anti-Mouse Immunoglobulins/HRP (cat. P0447, Agilent Technologies Denmark, Glostrup, DK). The β-actin antibody (cat. NB600-501, Novus Biologicals, Centennial, CO, USA) was used as a loading control. Protein bands were detected with Clarity Max Western ECL substrate. The band densitometry was analyzed with Fiji (ImageJ, Bethesda, MD, USA) and values were normalized to β-actin.

Western blot analysis of the activation of insulin signalling pathway was performed as described [3]. Myotubes were stimulated with or without 100 nmol/l insulin for 10 min at 37°C and lysed in ice-cold NP40-lysis buffer, pH 7.7 (10 mmol/l Tris, 150 mmol/l NaCl, 7 mmol/l EDTA, and 0.5% (vol/vol) NP-40) supplemented with protease and phosphatase inhibitors. Protein content was measured with BCA assay. Proteins were separated by SDS-PAGE, blotted onto PVDF membrane (Bio-Rad, Hercules, CA, USA), and probed with primary antibodies from Cell Signaling Technology (Danvers, MA, USA) against p-Akt-Ser^{473} (cat. #9271, 1:1000), total Akt (cat. #9272, 1:1000), p-AS160-Thr^{642} (cat. #4288, 1:1000), total AS160 (cat. #2447, 1:1000), p-GSK3β-Ser^{9} (cat. #9336, 1:1000), and total GSK3β (cat. #9315, 1:1000), followed by goat anti-rabbit HRP-conjugated secondary antibody (cat. 111-035-003, Jackson Laboratories, (West Grove, PA, USA)). Antibodies were diluted in EveryBlot Blocking Buffer (Bio-Rad). Protein bands were detected with an ECL 2 Western Blotting Substrate kit, visualized with ChemiDoc Touch imaging system (Bio-Rad), and quantified by using the Image Lab 6.0.1 software (Bio-Rad). Signal intensities were adjusted based on the stain-free total lane protein normalization method (Bio-Rad). Signal intensities of the phosphoproteins were normalised to the intensity of their corresponding total protein.

Metabolic assays

Glucose uptake was measured in triplicate by detecting the intracellular accumulation of 2-(1,2-[^{3}H])deoxy-D-glucose (final specific activity 3.7 GBq/mmol), as described [3]. Briefly, after the pretreatment with 10 μmol/l roxadustat or 0.1% (vol/vol) DMSO as control for 24 h primary human

myotubes were stimulated with or without 100 nmol/l insulin for 1 h, followed by the addition of radioactive glucose analog for 20 min. Cells were washed with ice-cold PBS and lysed with 0.4 mol/l NaOH following the neutralization with 4 mol/l HCl. Radioactivity (DPM) was detected with a scintillation counter. Cytochalasin B (50 µmol/l) was used to subtract the non-specific glucose uptake [4]. Values were adjusted to protein concentration measured with the Pierce BCA Protein Assay kit. Data (in pmol mg^{-1} min^{-1}) were expressed as a fold over basal control sample of each participant.

Glycogen synthesis was measured in triplicate by detecting D-[^{14}C]glucose (final specific activity 6.6 MBq/mmol) incorporation into glycogen [3]. Briefly, after the pretreatment with 10 µmol/l roxadustat or 0.1% DMSO as control for 24 h primary human myotubes were incubated with or without 100 nmol/l insulin together with radioactive glucose for 90 min. Cells were washed with ice-cold PBS and lysed with 0.03% (vol/vol) SDS. Glycogen was extracted by boiling for 30 min at 100°C together with 2.5 mg/ml glycogen carrier and precipitated with 94% ethanol at -20°C overnight. Precipitated glycogen was dissolved in H_2O, and radioactivity (DPM) was detected with a scintillation counter. Values were adjusted to protein concentration measured with BCA assay. Data (in nmol g^{-1} h^{-1}) were expressed as a fold over basal control sample of each participant.

Glycolysis was determined with Seahorse XFe96 analyzer (Agilent Technologies, CA, USA) in differentiated primary human myotubes under basal conditions using an XF Glycolytic Rate Assay kit (Agilent), according to the manufacturer´s instructions. Briefly, 10 000 cells/well were plated with 7-8 replicates per condition. After the pretreatment with 10 µmol/l roxadustat or 0.1% DMSO as control for 24 h, media was replaced with XF DMEM pH 7.4 (cat. #103575) containing 10 mmol/l glucose, 2 mmol/l glutamine, and 1 mmol/l sodium pyruvate, and incubated in CO_2-free 37°C incubator for 1 h. Stress compounds were injected according to the default assay program: 0.5 µmol/l antimycin A/rotenone and 50 mmol/l 2-deoxy-D-glucose. Raw data were adjusted to protein concentration measured with BCA assay. Glycolytic rate (proton efflux rate in pmol min^{-1} $µg^{-1}$) was expressed as a fold over control sample of each participant.

Mitochondrial respiration

Mitochondrial oxygen consumption rate was measured with a Seahorse XFe96 analyzer (Agilent Technologies) in differentiated primary human myotubes under basal conditions using XF Mito Stress Test Assay kit (Agilent Technologies, CA, USA), as described in the manufacturer´s instructions. Briefly, 10 000 cells/well were plated with 7-8 replicates per condition. After the

pretreatment with 10 µmol/l roxadustat or 0.1% DMSO as control for 24 h, media was replaced with XF DMEM pH 7.4 (cat. #103575) containing 5 mmol/l glucose, 2 mmol/l glutamine, and 1 mmol/l sodium pyruvate, and incubated in CO_2-free 37°C incubator for 1 h. Stress compounds were injected according to the default assay program; 1.5 µmol/l oligomycin to assess ATP-linked respiration, 1.5 µmol/l FCCP to assess maximal respiration, and 0.5 µmol/l antimycin A/rotenone to assess non-mitochondrial respiration. Raw data were adjusted to protein concentration measured with BCA assay. Data (in pmol min^{-1} μg^{-1}) were expressed as a fold over control sample of each participant.

ESM Table 1. Primers used in the quantitative PCR analyses.

Gene	Forward primer (5'->3')	Reverse primer (3'->5')
GBE1	GGAGATCGACCCGTACTTGAA	ACATCTGTGGACGCCAAATGA
GLUT1 (SLC2A1)	CTGAAGTCGCACAGTGAATA	TGGGTGGAGTTAATGGAGTA
HIF-P4H-2 (PHD2)	AGGCGATAAGATCACCTGGAT	TTCGTCCGGCCATTGATTTTG
HK1	GGACTGGACCGTCTGAATGT	ACAGTTCCTTCACCGTCTGG
HK2	ATTGTCCAGTGCATCGCGGA	AGGTCAAACTCCTCTCGCCG
LDHA	ACCCAGTTTCCACCATGATT	CCCAAAATGCAAGGAACACT
MCT4	CCATGCTCTACGGGACAGG	GCTTGCTGAAGTAGCGGTT
PDK1	CTATGAAAATGCTAGGCGTCTGT	TGGGATGGTACATAAACCACTTG
PFKL	CCCGAGGACGGCTGGGAGAA	GGGCTTCCCGTTGCGGTCAA
PPARA	TTCGCAATCCATCGGCGAG	CCACAGGATAAGTCACCGAGG
PPARG	TCTCTCCGTAATGGAAGACC	GCATTATGAGACATCCCCAC
TBP	GGGAGCTGTGATGTGAAGTTT	AAGGAGAACAATTCTGGGTTTG
VEGFA	AGGAGGAGGGCAGAATCATCA	ATGTCCACCAGGGTCTCGATTG

ESM Figures

ESM Figure 1.

Time course assay of the nuclear HIF1α (120 kDa) stabilization in primary human myotubes from men with normal glucose tolerance (n=2) exposed to 10 μmol/l roxadustat or 0.1% DMSO as control for 6 h, 24 h, and 48 h. Representative western blot images. HIF1α=hypoxia-inducible factor 1, p1=participant 1, p2=participant 2.

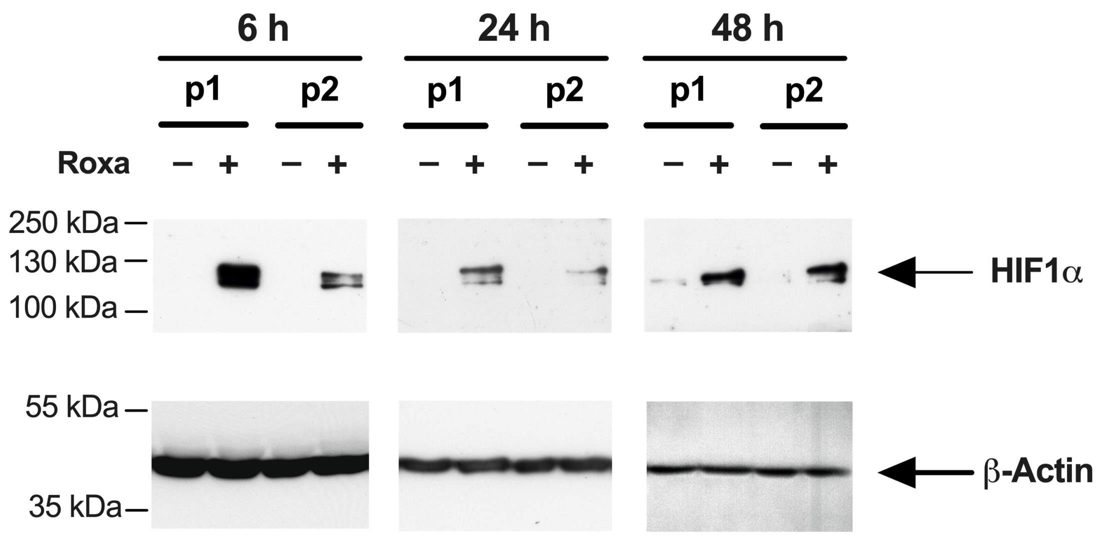

ESM Figure 2.

Time course assay of HIF target gene mRNA expression in primary human myotubes. Myotubes from three men with normal glucose tolerance were exposed to 10 µmol/l roxadustat or 0.1% DMSO as control for 6 h, 24 h, and 48 h. qPCR was used to detect the induction of HIF-responsive gene mRNAs. Data (in 2^-(ΔCt)) are expressed as boxes representing interleaved low-high, with line at mean. **p*<0.05, ***p*<0.01 for roxadustat vs control; analysed by two-way ANOVA with repeated measurements, with Holm–Šídák's post hoc test. ΔCt = difference in cycle threshold between the gene of interest and the reference gene.

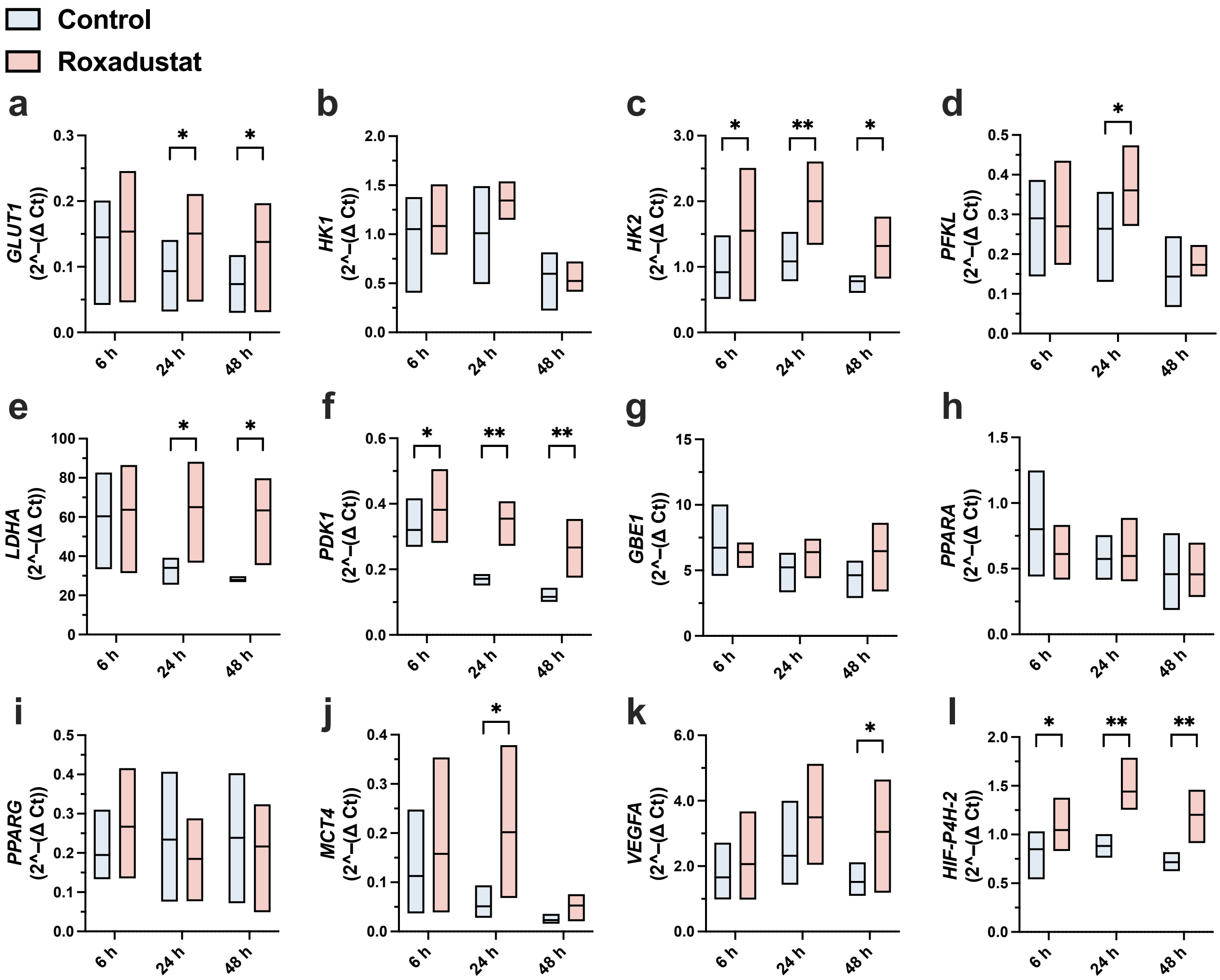

ESM Figure 3.

HIF target gene mRNA expression in roxadustat-treated myotubes. Primary human myotubes from men with NGT (n=5) or type 2 diabetes (n=5, consisting of three men with a low insulin response and two with a robust insulin response in glycogen synthesis assays) were exposed to 10 µmol/l roxadustat or 0.1% DMSO as control for 24 h. qPCR was used to detect the induction of HIF-responsive genes. NGT, normal glucose tolerance; T2D, type 2 diabetes. Data (in 2^-(ΔCt)) are expressed as mean ± SEM. *p<0.05, **p<0.01, ***p<0.001 for roxadustat vs control; analysed by two-way ANOVA with repeated measurements, with Holm–Šídák's post hoc test. ΔCt = difference in cycle threshold between the gene of interest and the reference gene.

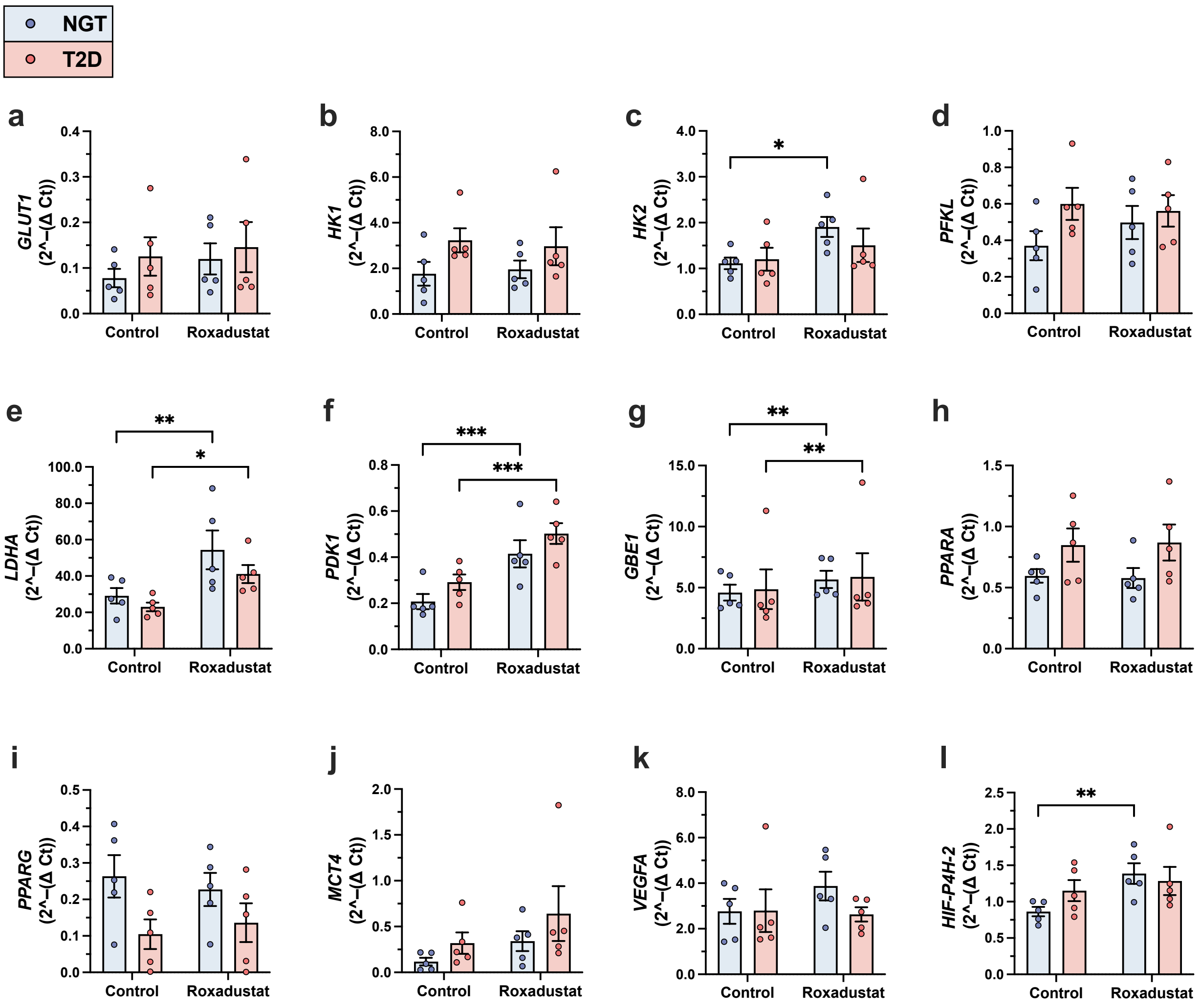

References

[1] Zhou M, Hou J, Li Y, et al. (2019) The pro-angiogenic role of hypoxia inducible factor stabilizer FG-4592 and its application in an in vivo tissue engineering chamber model. Scientific Reports 9(1): 6035. doi: 10.1038/s41598-019-41924-5

[2] Xie RY, Fang XL, Zheng XB, et al. (2019) Salidroside and FG-4592 ameliorate high glucose-induced glomerular endothelial cells injury via HIF upregulation. Biomed Pharmacother 118: 109175. doi: 10.1016/j.biopha.2019.109175

[3] Mäkinen S, Datta N, Rangarajan S, et al. (2023) Finnish-specific AKT2 gene variant leads to impaired insulin signalling in myotubes. Journal of Molecular Endocrinology 70(2): e210285. doi: 10.1530/JME-21-0285.

[4] Al-Khalili L, Chibalin AV, Kannisto K, et al. (2003) Insulin action in cultured human skeletal muscle cells during differentiation: assessment of cell surface GLUT4 and GLUT1 content. Cell Mol Life Sci 60(5): 991-998. doi: 10.1007/s00018-003-3001-3
